# Automatic classification of fetal heart rate based on a multi-scale LSTM network

**DOI:** 10.3389/fphys.2024.1398735

**Published:** 2024-06-12

**Authors:** Lin Rao, Jia Lu, Hai-Rong Wu, Shu Zhao, Bang-Chun Lu, Hong Li

**Affiliations:** ^1^ International Peace Maternity and Child Health Hospital, Shanghai Jiao Tong University School of Medicine, Shanghai, China; ^2^ Shanghai Key Laboratory of Embryo Original Diseases, Shanghai, China; ^3^ Key Laboratory of System Control and Information Processing, Ministry of Education of Shanghai Jiao Tong University, Shanghai, China

**Keywords:** fetal heart rate, long short-term memory, classification, multi time scale, artificial intelligence

## Abstract

**Introduction:**

Fetal heart rate monitoring during labor can aid healthcare professionals in identifying alterations in the heart rate pattern. However, discrepancies in guidelines and obstetrician expertise present challenges in interpreting fetal heart rate, including failure to acknowledge findings or misinterpretation. Artificial intelligence has the potential to support obstetricians in diagnosing abnormal fetal heart rates.

**Methods:**

Employ preprocessing techniques to mitigate the effects of missing signals and artifacts on the model, utilize data augmentation methods to address data imbalance. Introduce a multi-scale long short-term memory neural network trained with a variety of time-scale data for automatically classifying fetal heart rate. Carried out experimental on both single and multi-scale models.

**Results:**

The results indicate that multi-scale LSTM models outperform regular LSTM models in various performance metrics. Specifically, in the single models tested, the model with a sampling rate of 10 exhibited the highest classification accuracy. The model achieves an accuracy of 85.73%, a specificity of 85.32%, and a precision of 85.53% on CTU-UHB dataset. Furthermore, the area under the receiver operating curve of 0.918 suggests that our model demonstrates a high level of credibility.

**Discussion:**

Compared to previous research, our methodology exhibits superior performance across various evaluation metrics. By incorporating alternative sampling rates into the model, we observed improvements in all performance indicators, including ACC (85.73% vs. 83.28%), SP (85.32% vs. 82.47%), PR (85.53% vs. 82.84%), recall (86.13% vs. 84.09%), F1-score (85.79% vs. 83.42%), and AUC(0.9180 vs. 0.8667). The limitations of this research include the limited consideration of pregnant women’s clinical characteristics and disregard the potential impact of varying gestational weeks.

## 1 Introduction

Fetal heart rate (FHR) serves as an indicator of the fetal heart and central nervous system’s reaction to factors such as blood pressure, blood gases, and acid–base balance. In a clinical setting, FHR analysis can aid in the identification of fetal distress, placental abruption, chorioamnionitis, and other medical conditions (Sykes et al., 1983; Newton, 1993; Usui et al., 2007). FHR monitoring during labor is a valuable tool for detecting alterations in fetal heart rate patterns indicative of insufficient fetal oxygenation, enabling timely intervention by obstetricians to mitigate the risk of hypoxic injury or mortality. Electronic fetal monitoring (EFM) is currently recognized as a crucial modality for evaluating intrauterine fetal wellbeing and oxygenation levels (Sweha et al., 1999), owing to its ease of use and non-invasive nature. Consequently, EFM has emerged as an essential adjunctive screening method in obstetrics, with its utilization expanding in both antenatal and intrapartum settings.

The recording of dynamic changes in fetal heart rate can serve as an indirect indicator of fetal oxygen supply *in utero*, facilitating early detection of acute and chronic intrauterine hypoxia or asphyxia, thereby enhancing clinical efficiency. The cardiotocography (CTG) generated by EFM displays both FHR and uterine contractions, providing insights into their interplay (Alfirevic et al., 2017). Presently, three widely utilized clinical criteria exist for evaluating FHR monitoring. The first method of FHR interpretation discussed in academic literature is the nonstress test (NST) categorization outlined in the guidelines of the Society of Obstetricians and Gynecologists of Canada (SOGC), which classifies FHR as normal, atypical, and abnormal (Liston et al., 2007). The second approach is the three-tier FHR system jointly developed by the American College of Obstetricians and Gynecologists (ACOG), the Society for Maternal-Fetal Medicine (SMFM), and the National Institute of Children’s Health and Human Development (NICHD), which divides FHR into categories I, II, and III according to established criteria (Macones et al., 2008). The third source of guidance is the consensus guidelines on intrapartum fetal monitoring by the International Federation of Gynecology and Obstetrics (FIGO) and the National Institute for Health and Clinical Excellence (NICE), which categorize fetal monitoring into three classes: normal, suspicious, and pathological (Ayres-de Campos et al., 2015). The assessment of CTG basic features for each classification focuses on baseline, baseline variability, accelerations, and decelerations. However, despite standardized guidelines, discrepancies in recommendations and variations in obstetrician expertise contribute to significant diversity in observer interpretation of FHR.

In recent years, there has been an increasing integration of artificial intelligence (AI) technology in the healthcare sector, particularly in domains necessitating multifaceted inputs for evaluation and prompt decision-making. One notable application is in the realm of electronic fetal heart monitoring during labor and delivery. Using AI can minimize the variability among observers, enabling real-time interpretation of FHR data to prevent overlooking necessary interventions and enhance neonatal outcomes. Furthermore, AI provides a more standardized interpretation of the analysis of FHR monitoring findings.

Numerous researchers have endeavored to categorize FHR utilizing a blend of feature extraction and machine learning techniques. Georgoulas et al. (2006) conducted feature extractions in both time and frequency domains in conjunction with morphological features and applied a support vector machine (SVM) to classify the features. Spilka et al. (2012) utilized three types of features for classification, including 11 FIGO-like features, 14 heart rate variability-based features, and eight nonlinear features. Following dimensionality reduction, the classification model was trained using naive Bayes, SVM, and the C4.5 decision tree algorithm. Dash et al. (2014) incorporated additional features related to FHR responses to uterine contractions and subsequently conducted a comparative analysis of three generative models using SVM methods. Comert et al. (2016) utilized software to extract 21 features and implemented an extreme learning machine for data analysis. Spilka et al. (2017) advocated for sparse SVM classification, which offered the advantage of selecting a reduced number of features to detect various FHR patterns. In addition to traditional FHR features, techniques such as short-time Fourier transform (STFT), gray Level Co-occurrence matrix (GLCM) (Comert and Kocamaz, 2018), wavelet transform (Comert and Kocamaz, 2017), and common spatial pattern (CSP) (Alsaggaf et al., 2020) were employed to enhance classification performance.

All these methods were hindered by the requirement for feature extraction, which was typically done manually or with computer assistance. In response to this challenge, researchers introduced deep learning techniques to facilitate automatic feature extraction and classification. Convolutional neural networks (CNNs) have shown exceptional performance in image classification and have been extensively utilized in the medical field. Given that FHR signals are one-dimensional, researchers have explored various approaches to transform FHR signals into two-dimensional images, including STFT (Comert et al., 2019), continuous wavelet transform (CWT) (Zhao et al., 2019a), and recurrent plot (RP) (Zhao et al., 2019b). FHR analysis can be conducted using one-dimensional convolutional neural networks (1D-CNN) (Ismail Fawaz et al., 2019) as a time series method. Li et al. (2019) segmented 20-min FHR signals into 1–16 segments and applied 1D-CNN to analyze each segment, aggregating results through a voting mechanism. Cao et al. (Cao et al., 2023) employed a multimodal deep learning architecture (MMDLA) that integrates a CNN to extract high-level features from preprocessed cardiotocographic signals and maternal clinical data, thereby improving model performance. Zhou et al. (2023) proposed the trend-guided long convolution network (TGLCN), a deep learning methodology that integrates convolution kernel selection, residual structures, and attention mechanisms. Baghel et al. Baghel et al. (2022) utilized a Gaussian Butterworth band pass filter in conjunction with the CNN for the diagnosis of fetal acidosis. Furthermore, recurrent neural networks (RNNs), specifically long short-term memory (LSTM) networks, are crucial in FHR classification. Gao and Lu (2019) employed bidirectional LSTM (BiLSTM) for the segmental classification of FHR.

Although previous studies have made significant advances, certain challenges also persist, including imbalanced datasets affecting model performance and limited research on features at various time scales. To address these issues, this article introduces a multi-scale LSTM network. The article makes three key contributions: 1) Introducing a data augmentation methodology for time series to enhance datasets and address data imbalance. 2) Training LSTM models at different time scales through finetuning. 3) Proposing multi-scale LSTM networks to enhance model performance.

The subsequent sections of this article are organized as follows: Section 2 outlines the database utilized, the processing procedures applied, and the proposed methodology. Section 3 presents the experimental findings and compares them with previous studies. Section 4 provides a summary of the research and outlines potential future directions.

## 2 Methods

### 2.1 Dataset description

The dataset utilized in this study is the CTU-UHB database (Chuda´cˇek et al., 2014), an open-access repository comprising 552 recordings obtained at University Hospital in Brno (UHB) during the period of 2010–2012. Each recording is composed of two components: the cardiotocography (CTG) and clinical data. The CTG data are captured using three distinct methods: ultrasound Doppler probe, direct scalp measurement, or a hybrid approach. The CTG data encompass FHR and uterine contractions sampled at a rate of 4 Hz, resulting in four data points per second for each parameter.

**FIGURE 1:**
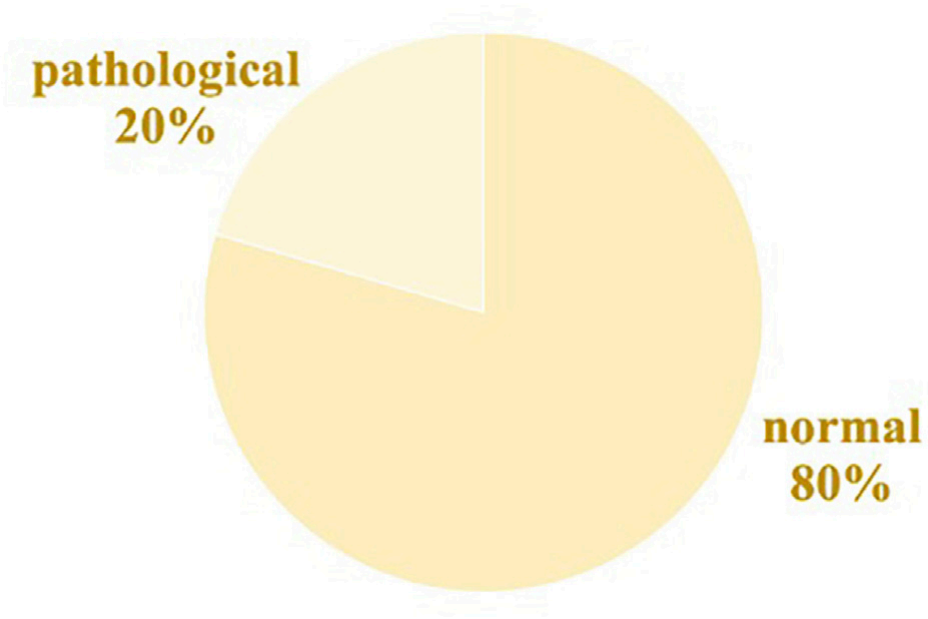
Class distribution.

The clinical data include information regarding fetal status and parameters concerning puerperal and newborn infants. Table 1 displays a portion of the clinical statistics obtained from the CTU-UHB database. Umbilical artery pH serves as a recognized marker for fetal acidemia, a condition associated with neonatal complications, such as multiple organ dysfunction in newborns (Sehdev et al., 1997; van den Berg et al., 1996). Studies have shown a relationship between FHR and variations in umbilical artery pH (Singh et al., 2021). Consequently, we employed the umbilical artery pH values from the clinical data to classify our dataset into two separate groups in Figure 1. In accordance with the established criterion that a pH value exceeding 7.15 signifies a normal condition, a total of 439 samples were classified as normal, and 113 samples were categorized as pathological based on their pH value (Comert et al., 2018).

**TABLE 1:** Patient and labor outcome statistics for the CTU-UHB cardiotocography database.

	Mean	Min	Max
Maternal age (years)	29.8	18	46
Parity	0.43	0	7
Gravidity	1.3	1	11
Gestational age (weeks)	40	37	43
pH	7.23	6.85	7.47
Base excess (BE, mmol/L)	−6.36	−26.8	−0.2
Base deficit in extracellular fluid (BDecf, mmol/L)	4.60	−3.40	
Apgar 1 min	8.26	1	10
Apgar 5 min	9.06	4	10
Neonatal weight(g)	3408	1970	4750

### 2.2 Data preprocessing

During the data collection process, missing signals and artifacts may arise in the original data due to external factors such as limitations in data acquisition by ultrasound probe and maternal and fetal movement, necessitating the preprocessing of data. The process is as follows:

(1) The original data are divided into 1-min segments, each containing 240 points. Then, the number of zero-value points $f_{0}$ are counted, and the data loss rate LR is calculated according to Eq. 1.

$$LR=\frac{f_{0}}{240}\times 100\%,$$ (1)

if LR ≥ 40%, this data segment will be discarded.

(2) When the FHR value is greater than 220 times per minute or less than 60 times per minute, it is treated as an abnormality due to poor contact with the acquisition device. The linear random interpolation method is used to replace the abnormal data. The formula of linear random interpolation is displayed according to Eq. 2.

$$f_{in}=\lambda f_{before}+\left(1-\lambda \right)f_{after,}$$ (2)

where λ is a random factor, and $f_{before}$ and $f_{after}$ are values before and after the missing point.

Due to too many missing signals in some recordings, the number of recordings in the dataset decreased to 550, with 439 normal recordings and 111 pathological recordings.

There are only 550 recordings in the dataset, and the ratio of normal recordings and pathological recordings is 4:1. The limited number of recordings and the ratio of normal to pathological readings can easily cause model overfitting. The length of recordings varies from 60 to 90 min. Under the instruction of obstetricians, we take 20-min signals to do further analysis. Thus, the dataset can be augmented by window slicing (Liang and Lu, 2023). The specific process is given as follows:

Step 1: For an FHR time series $T=\left\{t_{1},t_{2},\ldots ,t_{n}\right\}$ , choose the length of slicing window s and step length k ;

Step 2: Obtain the first slice with a window $T_{1}=\left\{t_{1},\ldots ,t_{s}\right\}$ ;

Step 3: Move the window to get $T_{2}=\left\{t_{k+1},\ldots ,t_{k+s}\right\}$ , ..., $T_{m}=\left\{t_{mk+1},\ldots ,t_{mk+s}\right\}$ and stop the process when mk+s>n .

**FIGURE 2:**
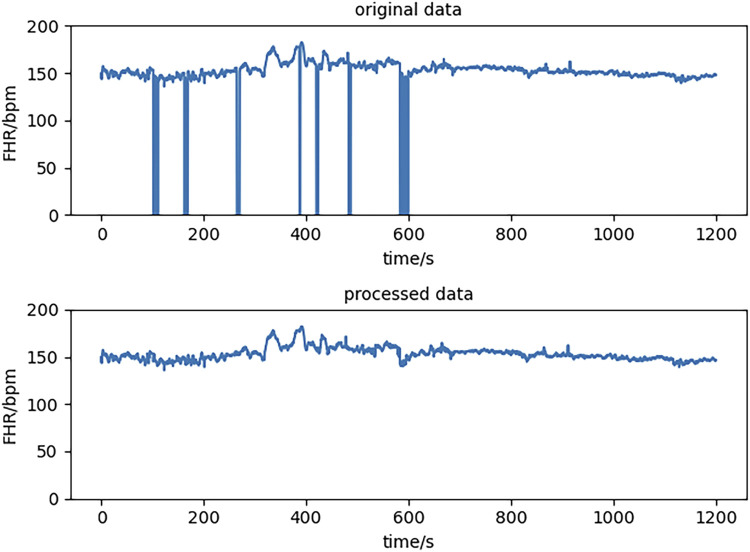
Data before and after preprocessing.

**FIGURE 3:**
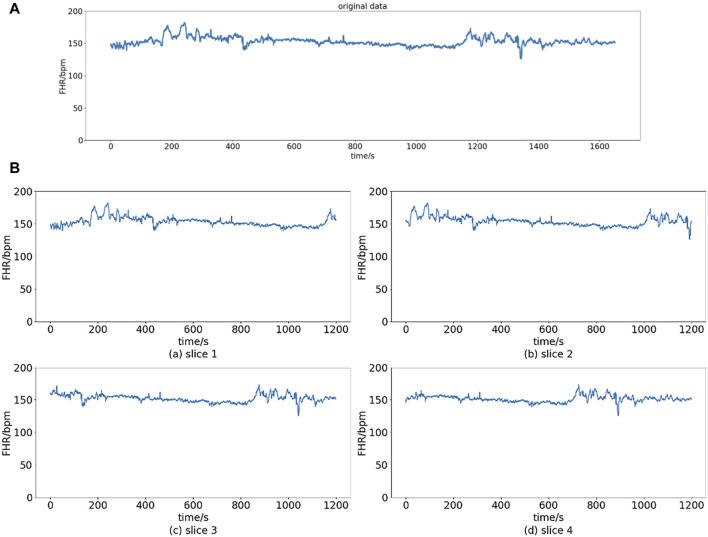
Slice operation. **(A)** Original data. **(B)** Slices of original data.

Figure 2 shows the signals before and after preprocessing. In this article, we chose s=4800 and k=600 , which implies generating 20-min samples with the beginnings of two adjacent samples that are 2.5 min apart. An example of a slice operation is shown in Figure 3.

After data augmentation, the number of normal samples increased to 6382 from 439, and the number of pathological samples increased to 1615 from 111. Because the two classes were still imbalanced, we chose 1,615 from 6,382 normal samples randomly to create a new dataset with all pathological samples.

### 2.3 LSTM networks

An LSTM is a special kind of RNN designed to solve the problem of long-term dependency (Hochreiter and Schmidhuber, 1997).

The workflow of the LSTM cell at time t is as follows: the hidden state of the previous moment and the input of the current moment enter the forget gate, input gate, and output gate for calculation and then update the cell state and hidden state. The input gate can decide what new information can be stored in the cell state, and the output gate determines what information can be output based on the cell state. The forget gate can decide what information will be discarded from the cell state. The calculation process is according to Eqs 3–8.

$$f_{t}=\sigma \left(W_{fh}h_{t-1}+W_{fx}x_{t}+b_{f}\right).$$ (3)

$$i_{t}=\sigma \left(W_{ih}h_{t-1}+W_{ix}x_{t}+b_{i}\right).$$ (4)

$$\tilde{c_{t}}=\tanh\left(W_{\tilde{ch}}h_{t-1}+W_{\tilde{cx}}x_{t}+b_{\tilde{c}}\right).$$ (5)

$$c_{t}=f_{t}\cdot c_{t-1}+i_{t}\cdot \tilde{c_{t}.}$$ (6)

$$o_{t}=\sigma \left(W_{oh}h_{t-1}+W_{ox}x_{t}+b_{o}\right).$$ (7)

$$h_{t}=o_{t}\cdot \tanh\left(c_{t}\right).$$ (8)

**FIGURE 4:**
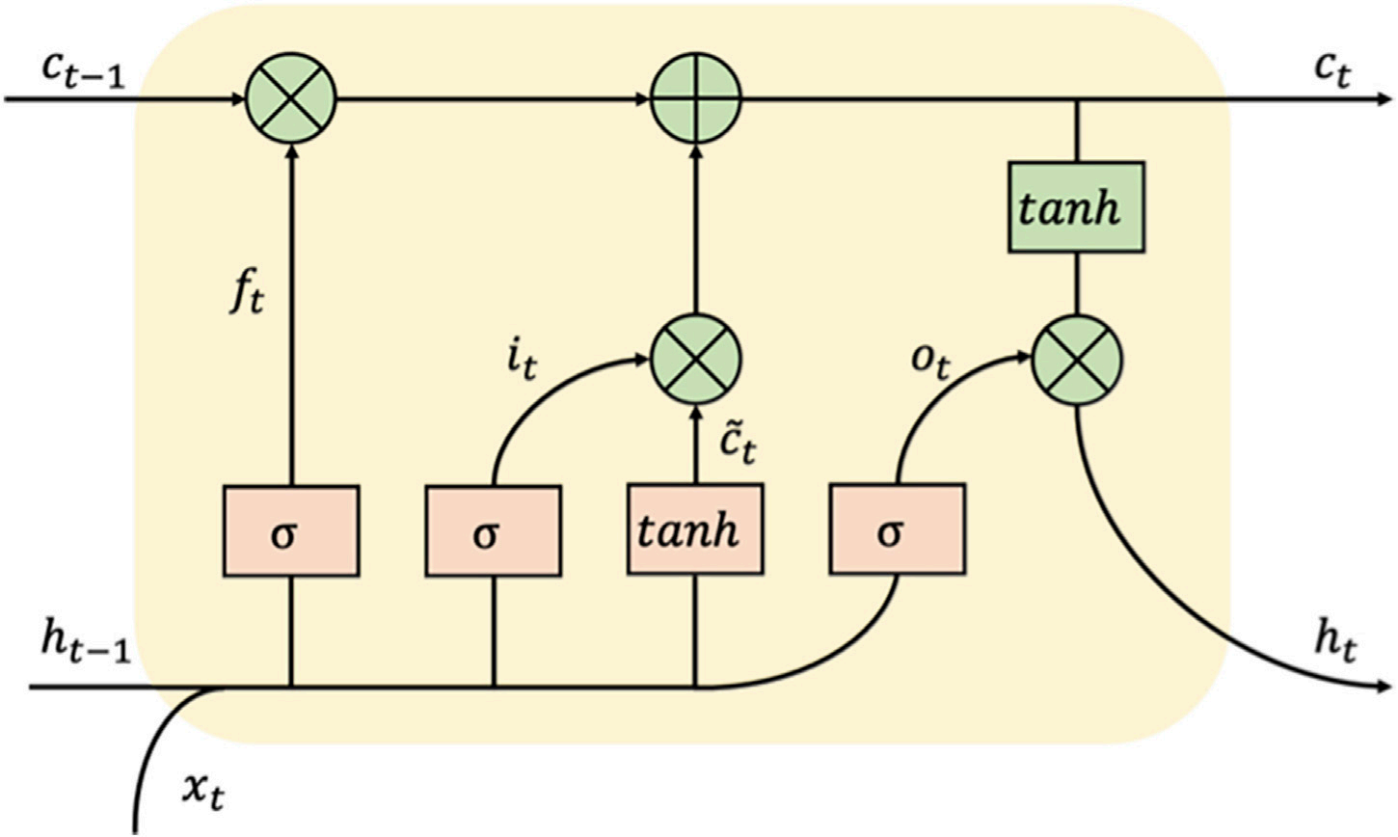
The architecture of an LSTM cell.

The architecture of LSTM cell is shown in Figure 4.

### 2.4 Multi-scale LSTM networks and voting mechanism

In clinical practice, obstetricians primarily utilize nonstress testing (NST) as the main modality for evaluating prenatal FHR. The SOGC (Liston et al., 2007) guidelines stipulate that interpreting NST results requires assessing various parameters, including baseline FHR, baseline variability, accelerations, and deceleration, each of which must be evaluated across different time intervals. For instance, the baseline FHR denotes the mean level of FHR over a 10-min period, excluding any accelerations, decelerations, or notable variability, and requires a minimum of 2 min of uninterrupted observation.

In contrast, acceleration and deceleration are typically evaluated within a time frame of less than 30 s. Consequently, the model must possess the capability to encompass both enduring characteristics that signify the general pattern in FHR data and fleeting characteristics that indicate minor fluctuations in specific areas. In accordance with this principle, we adopt the strategy of training numerous models by downsampling the data at varying frequencies. Downsampling is a prevalent technique in the processing of time series data. Downsampling facilitates the hybrid model in extracting data features across various time scales, thereby mitigating computational expenses and eliminating data redundancy (Liu et al., 2021).

**FIGURE 5:**
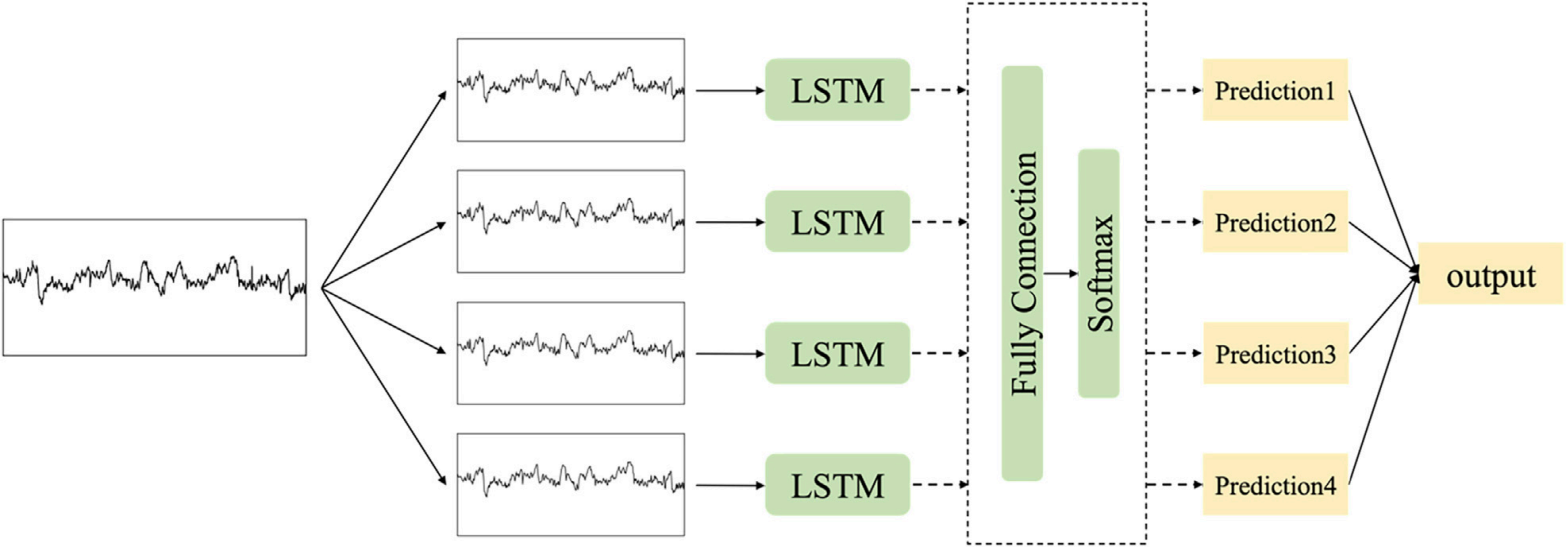
The architecture of multi-scale LSTM networks.

Subsequently, each dataset undergoes downsampling by distinct sampling intervals before being inputted into diverse time-scale LSTM models. These outputs of multi-scale models are aggregated using weights to yield the ultimate result, represented by the final result vector y denoting the probability of data belonging to each category. The computation process is according to Eqs 9, 10.

$$y=\Sigma_{i=1}^{n}\omega_{i}y_{i.}$$ (9)

$$\Sigma_{i=1}^{n}\omega_{i}=1,$$ (10)

where $y_{i}$ is the output vector of the i -th model and $\omega_{i}$ is the corresponding weight value of i -th model. The architecture of multi-scale LSTM networks is shown in Figure 5.

### 2.5 Evaluation index

The confusion matrix is a commonly utilized tool for assessing the efficacy of models in classification tasks (James et al., 2013). In the context of the binary classification discussed in this article, a confusion matrix with dimensions of two rows and two columns represents the frequency of four distinct prediction outcomes.

The metrics employed in our study include accuracy (ACC), specificity (SP), precision (PR), recall, F1-score, and area under the curve (AUC). ACC provides a comprehensive measure of the accuracy of predictions, while SP emphasizes the proportion of accurately identified negative samples. The constraints of electronic fetal monitoring contribute to a notable false positive rate in obstetric diagnoses. Inaccurate identification of pathological conditions may result in unwarranted medical interventions (Li et al., 2019). Therefore, it is imperative to consider precision and recall metrics, which evaluate the accuracy of positive predictions and the proportion of successfully detected positive samples. The F1-score represents the harmonic mean of PR and recall, while the quality index is calculated as the geometric mean of SP and sensitivity. The metrics mentioned above are calculated according to Eqs 11–15.

$$ACC=\frac{TP+TN}{TP+FP+TN+FN.}$$ (11)

$$SP=\frac{TN}{TN+FP}.$$ (12)

$$\mathrm{PR}=\frac{TP}{TP+FP}.$$ (13)

$$Recall=\frac{TP}{TP+FN}.$$ (14)

$$F1-score=2\cdot \frac{\mathrm{PR}\cdot Recall}{\mathrm{PR}+Recall}.$$ (15)

## 3 Experiments and results

### 3.1 Experimental settings

The experiment was carried out utilizing the PyTorch deep learning framework in Python, along with additional packages such as Numpy and Scikit-learn. The hardware configuration includes an Intel(R) Core (TM) i9-10900X CPU @ 3.70 Hz and an NVIDIA GeForce RTX 2080Ti.

The hybrid model is composed of two LSTM layers, three full connection layers, and an output layer, with each LSTM layer containing 512 hidden units. In order to address overfitting, a dropout rate of 0.2 is applied before the full connection layer. The output dimension is reduced to 2 through the full connection layers, with the final activation function being softmax for classification. The optimizer used is Adam, and the loss function employed is cross-entropy. To enhance the convergence of the network, we implemented a learning rate decay strategy during the training process consisting of 2,000 epochs. The initial learning rate was set at 0.001 and decreased by a factor of 10 after 500 and 1000 epochs.

The models were trained using a 10-fold cross-validation approach, where the dataset was partitioned into 10 subsets, each containing 323 samples. Nine subsets were utilized to train the model, while the remaining subset was used to test its performance. Following the training and testing of 10 models on the test set, the mean and standard deviation of the results were calculated.

### 3.2 Results analysis

Initially, the experiments were conducted to examine the impact of varying sampling rates on the efficacy of the model. The results presented in Table 2 indicate that the model exhibits optimal performance at a sampling rate of 10. Specifically, ACC and F1-score metrics demonstrate an improvement of approximately 5% compared to the next highest-performing model, while the SP and PR metrics show an enhancement of approximately 4.5%. The model’s performance improves with increasing sampling intervals, potentially due to its enhanced ability to discern between normal and pathological data by capturing long-term features. Furthermore, larger sampling intervals serve to diminish the impact of noise signals within the data.

**TABLE 2:** Comparison of the performance of different models.

Model	ACC (%)	SP (%)	PR (%)	Recall (%)	F1-score (%)	AUC
Sampling Rate = 4	74.49 ± 5.15	73.93 ± 4.33	74.15 ± 4.56	75.05 ± 6.54	74.57 ± 5.42	0.7699 ± 0.0552
Sampling Rate = 6	75.05 ± 4.39	73.68 ± 4.95	74.43 ± 4.53	76.41 ± 4.64	75.38 ± 4.34	0.7854 ± 0.0443
Sampling Rate = 8	78.39 ± 5.87	77.95 ± 6.51	78.22 ± 6.04	78.83 ± 6.56	78.47 ± 5.9	0.8193 ± 0.0626
Sampling Rate = 10	83.28 ± 4.37	82.47 ± 5.24	82.84 ± 4.68	84.09 ± 4.69	83.42 ± 4.24	0.8667 ± 0.0479
Multi-scale Model 1	85.73 ± 2.5	85.32 ± 3.68	85.53 ± 3.19	86.13 ± 3.1	85.79 ± 2.43	0.918 ± 0.0278
Multi-scale Model 2	84.92 ± 3.67	84.51 ± 5.06	84.78 ± 4.42	85.33 ± 4.01	85 ± 3.54	0.914 ± 0.0316
Multi-scale Model 3	84.18 ± 3.5	87.86 ± 5.15	87.11 ± 4.67	80.5 ± 5.12	83.56 ± 3.67	0.8992 ± 0.0375

**FIGURE 6:**
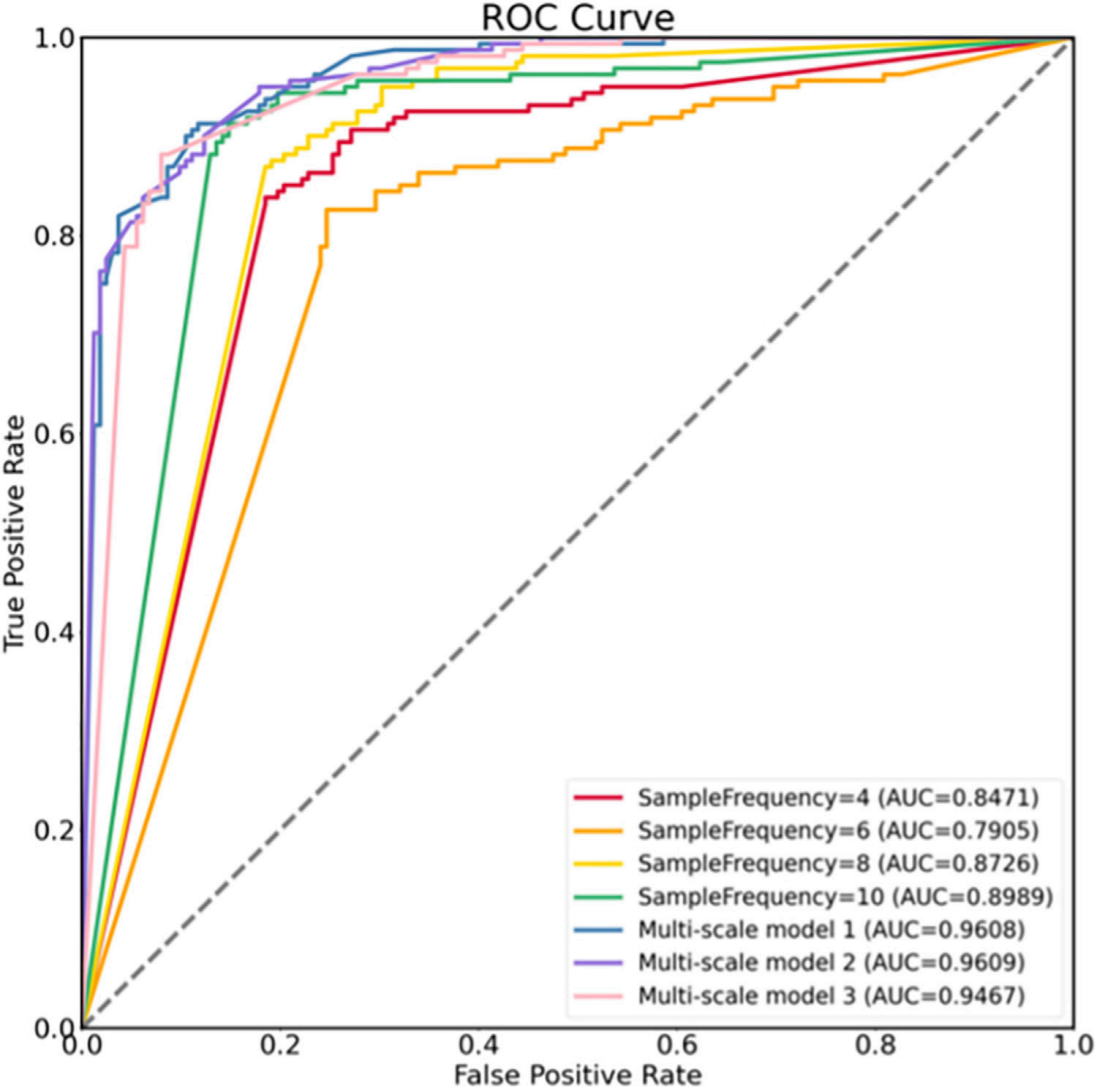
ROCs of different models.

Three different multi-scale models were constructed by manipulating the quantity and magnitude of the component models. Multi-scale Model 1 comprises four sampling rates: 4, 6, 8, 10. Multi-scale Model 2 utilizes models with sampling rates of 4, 8, and 10, whereas multi-scale Model 3 exclusively integrates models with sampling rates of 8 and 10. The superior performance of all multi-scale models over the single models is evident in Table 2, indicating that the incorporation of multi-scale features aids in mitigating overfitting to some degree and enhances categorization accuracy. Multi-scale Model 1 demonstrates superior performance on ACC, recall, F1-score, and AUC, suggesting that incorporating diverse time-scale features enhances classification accuracy. Conversely, Model 3 exhibits higher SP and PR but comparatively lower performance on other evaluation criteria. The ROC curve depicted in Figure 6 illustrates the discriminative capabilities of single models *versus* multi-scale models, with the latter showcasing an enhanced ability to distinguish between two classes.

### 3.3 Discussion

In this research, we introduce a multi-scale LSTM model integrated with models that target various time scales. Experimental analyses were carried out on both single and multi-scale models. The results demonstrate that multi-scale LSTM models outperform regular LSTM models in various performance metrics. Specifically, among the single models tested, the model with a sampling rate of 10 exhibited the highest classification accuracy. Incorporating alternative sampling rates into the model resulted in enhancements across all performance indicators, including ACC (85.73% vs. 83.28%), SP (85.32% vs. 82.47%), PR (85.53% vs. 82.84%), recall (86.13% vs. 84.09%), F1-score (85.79% vs. 83.42%), and AUC (0.9180 vs. 0.8667).

To illustrate the importance of our model, the outcomes of both machine learning (Comert et al., 2018; O’Sullivan et al., 2021; Ben Barek et al., 2023) and deep learning approaches (Liu et al., 2021; Singh et al., 2021) utilizing the identical dataset are presented in Table 3. Our model exhibits superior performance in terms of ACC, SP, PR, recall, and AUC compared to the aforementioned machine learning methods (Liu et al., 2021; Singh et al., 2021). Furthermore, when compared to a specific model (Liu et al., 2021), our model demonstrates notably higher levels of ACC, SP, and recall. It is worth noting that the model discussed (Singh et al., 2021) achieves an ACC of 69.6%, potentially attributed to the limitations of CNNs in capturing temporal features effectively. This observation suggests that our model possesses enhanced classification capabilities.

**TABLE 3:** Comparison of the proposed model with previous work.

References	Method	ACC (%)	SP (%)	PR (%)	Recall (%)	F1-score (%)	AUC
Comert et al. (2018)	EMD + DWT + SVM	67.00	67.26	\	57.42	\	\
O’Sullivan et al. (2021)	ARMA + SVM	83.3	77.7	\	82.6	\	0.809
Liu et al. (2021)	CNN-BiLSTM + Attention, DWT	71.71 ± 8.61	70.81 ± 12.20	\	75.23 ± 9.58	\	\
Singh et al. (2021)	HoloViz + CNN	69.6	\	63	70	66	\
Ben Barek et al. (2023)	LR	\	\	\	\	\	0.74
Ours	Multi-scale LSTM	85.73 ± 2.5	85.32 ± 3.68	85.53 ± 3.19	86.13 ± 3.1	85.79 ± 2.43	0.918 ± 0.0278

In conclusion, the proposed model demonstrates enhanced performance in the classification of FHR. This model offers several advantages, including directly classifying FHR signals without the need for complex feature extraction processes and ensuring immediate discrimination. Additionally, incorporating various time-scale signals enables the model to effectively learn both long-term and short-term features, thereby optimizing overall performance.

## 4 Conclusion

In this study, a multi-scale LSTM model was developed for the automatic classification of FHR. The publicly available CTU-UHB database was utilized for this purpose. Following data preprocessing and enhancement, FHR signals were employed as input for the models. The proposed model demonstrated the ability to identify pathological FHR patterns. Experimental results indicate that our model outperforms common LSTM models and previous research efforts in terms of various metrics. Specifically, the model achieved an accuracy, specificity, and precision of 89.78%, 91.36%, and 91.03%, respectively. Our work presents significant contributions in utilizing the LSTM model for extracting hidden features from FHR signals, eliminating the need for manual feature extraction. Additionally, incorporating various time-scale features enhances the performance of the models. Ultimately, our model facilitates intelligent recognition of FHR, aiding obstetricians in identifying abnormal FHR patterns and supporting timely treatment interventions.

Nevertheless, it is important to acknowledge the limitations of our research. First, the clinical characteristics of pregnant women, including maternal age and weight, can significantly influence the classification results and should be taken into consideration. Second, the data in the CTU-UHB dataset were gathered 90 min prior to delivery, potentially overlooking the impact of varying gestational weeks on fetal heart rate patterns, particularly around 32 weeks. Moving forward, we plan to establish partnerships with medical facilities to expand our dataset by incorporating additional fetal heart rate, uterine contraction, and clinical information. Further analysis of additional features should be conducted during the model construction process, and adjustments to the model structure should be made in order to enhance classification accuracy.

## Data Availability

The raw data supporting the conclusion of this article will be made available by the authors, without undue reservation.

## References

[B1] Alfirevic Z. Gyte G. M. Cuthbert A. Devane D. (2017). Continuous cardiotocography (ctg) as a form of electronic fetal monitoring (efm) for fetal assessment during labour. Cochrane Database Syst. Rev. 2019 (5), CD006066. 10.1002/14651858.CD006066.pub3 16856111

[B2] Alsaggaf W. Comert Z. Nour M. Polat K. Brdesee H. Tog˘ac¸ar M. (2020). Predicting fetal hypoxia using common spatial pattern and machine learning from cardiotocography signals. Appl. Acoust. 167, 107429. 10.1016/j.apacoust.2020.107429

[B3] Ayres-de Campos D. Spong C. Y. Chandraharan E. Panel F. I. F. M. E. C. (2015). Figo consensus guidelines on intrapartum fetal monitoring: cardiotocography. Int. J. Gynecol. Obstetrics 131 (1), 13–24. 10.1016/j.ijgo.2015.06.020 26433401

[B4] Baghel N. Burget R. Dutta M. K. (2022). 1d-fhrnet: automatic diagnosis of fetal acidosis from fetal heart rate signals. Biomed. Signal Process. Control 71, 102794. 10.1016/j.bspc.2021.102794

[B5] Ben Barek I. Jauvion G. Vitrou J. Holmstro E. Koskas M. Ceccaldi P.-F. (2023). DeepCTG® 1.0: an interpretable model to detect fetal hypoxia from cardiotocography data during labor and delivery. Front. Pediatr. 11, 1190441. 10.3389/fped.2023.1190441 37397139 PMC10311205

[B6] Cao Z. Wang G. Xu L. Li C. Hao Y. Chen Q. (2023). Intelligent antepartum fetal monitoring via deep learning and fusion of cardiotocographic signals and clinical data. Health Inf. Sci. Syst. 11 (1), 16. 10.1007/s13755-023-00219-w 36950107 PMC10025176

[B7] Chuda cek V. Spilka J. Bursa M. Janku P. Hruban L. Huptych M. (2014). Open access intrapartum ctg database. BMC Pregnancy Childbirth 14 (1), 16. 10.1186/1471-2393-14-16 24418387 PMC3898997

[B8] Comert Z. Yang Z. Velappan S. Boopathi A. M. Kocamaz A. F. (2018). “Performance evaluation of empirical mode decomposition and discrete wavelet transform for computerized hypoxia detection and prediction,” in 2018 26th Signal Processing and Communications Applications Conference (SIU), Izmir, Turkey, May, 2018, 1–4.

[B9] Comert Z. Kocamaz A. F. (2017). “Using wavelet transform for cardiotocography signals classification,” in 2017 25th Signal Processing and Communications Applications Conference (SIU), Antalya, Turkey, May, 2017, 1–4.

[B10] Comert Z. Kocamaz A. F. (2018). Open-access software for analysis of fetal heart rate signals, Biomedical. Signal Process. Control 45, 98–108. 10.1016/j.bspc.2018.05.016

[B11] Comert Z. Kocamaz A. F. (2019). “Fetal hypoxia detection based on deep convolutional neural network with transfer learning approach,” in Software engineering and algorithms in intelligent systems. Editor Silhavy R. (Cham: Springer International Publishing), 239–248.

[B12] Comert Z. Kocamaz A. F. Gu¨ngo¨r S. (2016). “Cardiotocography signals with artificial neural network and extreme learning machine,” in 2016 24th Sig- nal Processing and Communication Application Conference (SIU), Zonguldak, Turkey, May, 2016, 1493–1496.

[B13] Comert Z. Kocamaz A. F. Subha V. (2018). Prognostic model based on image-based time-frequency features and genetic algorithm for fetal hypoxia assessment. Comput. Biol. Medicine99 99, 85–97. 10.1016/j.compbiomed.2018.06.003 29894897

[B15] Dash S. Quirk J. G. Djuric´ P. M. (2014). Fetal heart rate classification using generative models. IEEE Trans. Biomed. Eng. 61 (11), 2796–2805. 10.1109/TBME.2014.2330556 24951678

[B16] Gao W. Lu Y. (2019). “Fetal heart baseline extraction and classification based on deep learning,” in 2019 International Conference on Information Technology and Computer Application (ITCA), Guangzhou, China, December, 2019, 211–216.

[B17] Georgoulas G. Stylios D. Groumpos P. (2006). Predicting the risk of metabolic acidosis for newborns based on fetal heart rate signal classification using support vector machines. IEEE Trans. Biomed. Eng. 53 (5), 875–884. 10.1109/TBME.2006.872814 16686410

[B18] Hochreiter S. Schmidhuber J. (1997). Long short-term memory. Neural Comput. 9 (8), 1735–1780. 10.1162/neco.1997.9.8.1735 9377276

[B19] Ismail Fawaz H. Forestier G. Weber J. Idoumghar L. Muller P.-A. (2019). Deep learning for time series classification: a review. Data Min. Knowl. Discov. 33 (4), 917–963. 10.1007/s10618-019-00619-1

[B20] Ito E. H. Nagasaki S. Kotaki H. Shimabukuro M. Sakuma J. Takano M. (2022). Optimal duration of cardiotocography assessment using the ipreface score to predict fetal acidemia. Sci. Rep. 12, 13064. 10.1038/s41598-022-17364-z 35906383 PMC9338067

[B21] James G. Witten D. Hastie T. Tibshirani R. (2013) An introduction to statistical learning. New York: Springer.

[B22] Li J. Chen Z.-Z. Huang L. Fang M. Li B. Fu X. (2019). Automatic classification of fetal heart rate based on convolutional neural network. IEEE Internet Things J. 6 (2), 1394–1401. 10.1109/jiot.2018.2845128

[B23] Liang H. Lu Y. (2023). A cnn-rnn unified framework for intrapartum cardiotocograph classification. Comput. Methods Programs Biomed. 229, 107300. 10.1016/j.cmpb.2022.107300 36566652

[B24] Liston R. Sawchuck D. Young D. Brassard N. Campbell K. Davies G. (2007). Fetal health surveillance: antepartum and intrapartum consensus guideline. J. Obstetrics Gynaecol. Can. 29 (9), S3–S56. 10.1016/S1701-2163(16)32615-9 17845745

[B25] Liu M. Lu Y. Long S. Bai J. Lian W. (2021). An attention-based cnn-bilstm hybrid neural network enhanced with features of discrete wavelet transformation for fetal acidosis classification. Expert Syst. Applications186 186, 115714. 10.1016/j.eswa.2021.115714

[B26] Macones G. A. Hankins G. D. V. Spong C. Y. Hauth J. Moore T. (2008). The 2008 national institute of child health and human development workshop report on electronic fetal monitoring: update on definitions, interpretation, and research guidelines. J. Obstetric, Gynecol. &Neonatal Nurs. 37 (5), 510–515. 10.1111/j.1552-6909.2008.00284.x 18761565

[B27] Newton E. (1993). Chorioamnionitis and intraamniotic infection. Clin. obstetrics Gynecol. 36 (4), 795–808. 10.1097/00003081-199312000-00004 8293582

[B28] O Sullivan M. Gabruseva T. Boylan G. O’Riordan M. Lightbody G. Marnane W. (2021). “Classification of fetal compromise during labour: signal processing and feature engineering of the cardiotocograph,” in 2021 29th European Signal Processing Conference (EUSIPCO), Dublin, Ireland, August, 2021, 1331–1335.

[B29] Sehdev H. M. Stamilio D. M. Macones G. A. Graham E. Morgan M. A. (1997). Predictive factors for neonatal morbidity in neonates with an umbilical arterial cord pH less than 7.00. Am. J. Obstetrics Gynecol. 177 (5), 1030–1034. 10.1016/s0002-9378(97)70008-5 9396887

[B30] Singh H. D. Saini M. Kaur J. (2021). Fetal distress classification with deep convolutional neural network. Curr. Women’s Health Rev. 17 (1), 60–73. 10.2174/1573404816999200821162312

[B31] Spilka J. Chuda´cˇek V. Koucky´ M. Lhotska´ L. Huptych M. Janku˚ P. (2012). Using nonlinear features for fetal heart rate classification. Biomed. Signal Process. Control 7 (4), 350–357. 10.1016/j.bspc.2011.06.008

[B32] Spilka J. Frecon J. Leonarduzzi R. Pustelnik N. Abry P. Doret M. (2017). Sparse support vector machine for intrapartum fetal heart rate classification. IEEE J. Biomed. Health Inf. 21 (3), 664–671. 10.1109/JBHI.2016.2546312 27046884

[B33] Sweha A. Hacker T. Nuovo J. (1999). Interpretation of the electronic fetal heart rate during labor. Am. Fam. physician 59 (9), 2487–2500. 10323356

[B34] Sykes G. S. Molloy P. M. Johnson P. Stirrat G. M. Turnbull A. C. (1983). Fetal distress and the condition of newborn infants. BMJ 287 (6397), 943–945. 10.1136/bmj.287.6397.943 6412897 PMC1549216

[B35] Usui R. Matsubara S. Ohkuchi A. Kuwata T. Watanabe T. Izumi A. (2007). Fetal heart rate pattern reflecting the severity of placental abruption. Archives Gynecol. Obstetrics 277 (3), 249–253. 10.1007/s00404-007-0471-9 17896112

[B36] van den Berg P. P. Nelen W. L. Jongsma H. W. Nijland R. Kolle´e L. A. Nijhuis J. G. (1996). Neonatal complications in newborns with an umbilical artery pH < 7.00. Am. J. Obstetrics Gynecol. 175 (5), 1152–1157. 10.1016/s0002-9378(96)70021-2 8942481

[B37] Zhao Z. Deng Y. Zhang Y. Zhang Y. Zhang X. Shao L. (2019a). Deepfhr: intelligent prediction of fetal acidemia using fetal heart rate signals based on convolutional neural network. BMC Med. Inf. Decis. Mak. 19 (1), 286. 10.1186/s12911-019-1007-5 PMC6937790 31888592

[B38] Zhao Z. Zhang Y. Comert Z. Deng Y. (2019b). Computer-aided diagnosis system of fetal hypoxia incorporating recurrence plot with convolutional neural network. Front. Physiology 10, 255. 10.3389/fphys.2019.00255 PMC6422985 30914973

[B39] Zhou Z. Zhao Z. Zhang X. Zhang X. Jiao P. Ye X. (2023). Identifying fetal status with fetal heart rate: deep learning approach based on long convolution. Comput. Biol. Med. 159, 106970. 10.1016/j.compbiomed.2023.106970 37105114

